# Mouse models in COVID-19 research: analyzing the adaptive immune response

**DOI:** 10.1007/s00430-022-00735-8

**Published:** 2022-06-04

**Authors:** Sabrina Clever, Asisa Volz

**Affiliations:** 1 Institute of Virology, University of Veterinary Medicine Hannover, Hannover, Germany; 2 Research Center for Emerging Infections and Zoonoses, University of Veterinary Medicine Hannover, Hannover, Germany

**Keywords:** COVID-19, SARS-CoV-2, Mouse models, Specific T cells

## Abstract

The emergence of SARS-CoV-2, the severe acute respiratory syndrome coronavirus type 2 causing the COVID-19 pandemic, resulted in a major necessity for scientific countermeasures. Investigations revealing the exact mechanisms of the SARS-CoV-2 pathogenesis provide the basis for the development of therapeutic measures and protective vaccines against COVID-19. Animal models are inevitable for infection and pre-clinical vaccination studies as well as therapeutic testing. A well-suited animal model, mimicking the pathology seen in human COVID-19 patients, is an important basis for these investigations. Several animal models were already used during SARS-CoV-2 studies with different clinical outcomes after SARS-CoV-2 infection. Here, we give an overview of different animal models used in SARS-CoV-2 infection studies with a focus on the mouse model. Mice provide a well-established animal model for laboratory use and several different mouse models have been generated and are being used in SARS-CoV-2 studies. Furthermore, the analysis of SARS-CoV-2-specific T cells during infection and in vaccination studies in mice is highlighted.

## COVID-19 pandemic and its countermeasures

The coronavirus disease 2019 (COVID-19) pandemic is still ongoing and research regarding its countermeasures is of high value. The causative agent of this disease, the severe acute respiratory syndrome coronavirus-2 (SARS-CoV-2), can induce a variety of rather mild symptoms, including pneumonia [1] and several different symptoms like fever, cough, dyspnea, myalgia, or even cardiac dysfunctions [2, 3]. Nonetheless, in severe cases, acute respiratory distress syndrome (ARDS) and septic shock can occur [4]. Furthermore, impairment of other organ systems has also been described during COVID-19 and those severe symptoms occur more prominently in higher risk patients with for example diabetes, obesity, or chronic respiratory diseases. Several steps of virus-induced pathogenesis have been rudimentarily illuminated but still little is known about its exact mechanisms [5–7]. Countermeasures to fight COVID-19 have been rapidly developed, starting with hygienic measurements up to authorized vaccines, which are already frequently used. Nevertheless, those vaccines still can be improved and have to be adapted to new upcoming variants of SARS-CoV-2. Additionally, it should be borne in mind that health-impaired people are unable to be vaccinated or build a protective immune response [8]. Thus, understanding the complete mechanisms of the pathogenesis for the development of therapeutics and also optimized vaccines is important. For this, the usage of appropriate animal models in research studies is inevitable. Human sample collection, like blood or saliva, for example, is possible and provides a lot of information [9, 10]. Due to the high number of infected persons, many samples can be collected, which is often a limitation for other human disease research. Results can be more easily transferred to human therapeutic and vaccination development, but the results from those samples merely display a small part of the complete immune response. Analysis of important immunological organs plays a key role in pathogenesis and vaccination studies, which can only be analyzed in animal models. Human vaccination studies are also in the need of pre-clinical testing in animal models because of unexpected severe side-effects [11]. One controversial aspect are human infection studies, since they could improve and accelerate the process of research but pose ethical issues regarding severe disease courses after an intentional application of a pathogen [12].

The choice of a well-suited animal model for the respective pathogen is crucial, since not every animal mimics the disease patterns in such a way that these can be compared to the human disease course. For COVID-19, several different animal models have been taken into consideration. Most prominently, hamsters, ferrets, and non-human primates are used, since they are naturally susceptible to SARS-CoV-2. The most common animal species for scientific investigations and also a prominent species in COVID-19 research are mice. These different animal models will be discussed in the following chapters.

## Animal models for SARS-CoV-2

The animal models for SARS-CoV-2 research and pre-clinical trials should mimic important aspects of the COVID-19 disease for the investigations. One key player for infection is the receptor that the virus uses for cell entry. Previous investigations already showed that similar to SARS-CoV, SARS-CoV-2 also binds to the hACE2 receptor [13]. There are several other species, for example, cats, dogs, or minks, that are also susceptible to SARS-CoV-2, but they have differences in the ACE2 receptors sequences. Moreover, the previous studies revealed evidence that the human ACE2 receptor has the highest binding affinity to SARS-CoV-2 compared to other animal species [14–16]. Those differences should be taken into consideration during animal SARS-CoV-2 infection studies. For these studies, several animal models were under discussion, like for example non-human primates, hamsters, ferrets, minks, and mice. They provide several different advantages, especially concerning the different scientific questions. In the following, the animal models will be discussed in more detail and a summary can be seen in Table 1.

**Table 1:** Animal models for COVID-19. Listed are different animal species (monkey, hamster, ferret, mink, and mouse) involved in SARS-CoV-2 infection studies

Infection studies					
Species	SARS-CoV-2 Isolate	Infection Route	Dosage	Results	Reference
*Monkey*					
Rhesus macaques	USA-WA1/2020	Ocular, i.n., i.t	1.05 × 10^6^ PFU	Clinical signs of viral infection, mild-to-moderate pneumonitis and extra-pulmonary pathologies; recovery after 2 weeks	Singh et al. Nat. Microbiol. 2021
	BetaCoV/Wuhan/IVDC‐HB‐01/2020|EPI_ISL_402119	i.t	10^6^ TCID_50_	Old animals: more viral replication in respiratory tract; severe interstitial pneumonia	Yu et al. Animal Model Exp. Med. 2020
	SARS-CoV-2 strain 107 (Guangdong Provincial CDC, Guangdong, China)	i.t	1 × 10^7^ TCID_50_	Young animals: impaired respiratory function and quick recovery; aged animals: delayed severe cytokine storm	Song et al. Zool. Res. 2020
	2019-nCoV/USA-WA1/2020	Aerosol; oral, i.n., i.t., conjunctival	2 × 10^3^; 3.61 × 10^6^ PFU	Minor clinical, radiographic, and histopathologic changes	Blair et al. Am. J. Pathol. 2021
	2019-nCoV/USA-WA1/2020 (Lot R4717)	Aerosol	3.84 × 10^4^ PFU	Respiratory abnormalities and viral shedding; thrombocytopenia	Johnston et al. PLoS One 2021
	nCoV-WA1–2020	i.n. + i.t. + oral + ocular	4 × 10^5^ TCID_50_	Viral replication in the respiratory tract; pulmonary infiltrates; moderate disease	Munster et al. Nature, 2020
Cynomolgus macaques	2019-nCoV/USA-WA1/2020 (Lot R4717)	Aerosol	3.84 × 10^4^ PFU	Respiratory abnormalities and viral shedding; fever, alveolar fibrosis	Johnston et al. PLoS One 2021
	BetaCoV/Munich/BavPat1/2020	i.n. + i.t	10^6^ TCID_50_	No clinical signs, virus titer in respiratoy tract	Rockx et al. Science, 2020
African green monkeys	2019-nCoV/USA-WA1/2020	Aerosol; oral, i.n., i.t., conjunctival	2 × 10^3^; 3.61 × 10^6^ PFU	Acute respiratory distress syndrome (ARDS) in 2 aged AGMs	Blair et al. Am. J. Pathol. 2021
	SARS-CoV-2/INMIl-Isolate/2020/Italy	i.n. + i.t	4.6 × 10^5^ PFU	Viral replication in mucosal samples, respiratory disease, inflammation and coagulopathy in blood and tissues	Woolsey et al. Nat. Immunol., 2021
	2019-nCoV/USA-WA1/2020 (Lot R4717)	Aerosol	3.84 × 10^4^ PFU	Respiratory abnormalities and viral shedding, thrombocytopenia, alveolar fibrosis	Johnston et al. PLoS One 2021
*Hamster*					
	BetaCoV/Hong Kong/VM20001061/2020 virus	i.n	8 × 10^4^ TCID_50_	Viral replication in the respiratory tract (gone at 7dpi), efficient transmission between animals, inflammatory cell infiltration in lung	Sia et al. Nature, 2020
	UT-NCGM02	i.n. and ocular	10^5.6^ PFU, 10^3^ PFU	Viral replication in the lung, severe pathological lung lesions	Imai et al. PNAS, 2020
	Canada/ON/VIDO-01/2020	i.n	10^5^ TCID_50_	Viral replication in the respiratory tract, inflammation in the lung, kidney, liver, and heart, increased inflammatory cytokine level	Francis et al. PLoS Pathog., 2021
	Isolated from the nasopharyngeal aspirate specimen of a COVID-19 patient (Chan et al. J.Clin.Microbiol, 2020)	i.n	10^5^ PFU	Viral replication in the lung; diffuse alveolar damage with followed tissue repair, effective transmission between animals	F-W Chan et al. Clin. Infect. Dis., 2020
*Ferret*					
	SARS-CoV-2/F13/environment/2020/Wuhan (F13-E); SARS-CoV-2/CTan/human/2020/Wuhan (CTan-H)	i.n	10^5^ PFU	Viral replication in upper respiratory tract, minor transmission	Shi et al. Science, 2020
	SARS-CoV-2 Victoria/01/2020	i.n	5 × 10^6^, 5 × 10^4^, 5 × 10^2^ PFU	Viral replication in upper respiratory tract, mild clinical disease	Ryan et al. Nat. Commun., 2021
	SARS-CoV-2 2019_nCoV Muc-IMB-1	oculo-oronasal	10^5^ TCID_50_	Viral replication in the upper respiratory tract, no clinical signs, transmission to naive ferrets	Schlottau et al. Lancet Microbe, 2020
	USA-WA1/2020	i.n	5 × 10^4^ PFU	Low viral replication in the upper respiratory tract but mixed leukocyte infiltration with cytokine production	Blanco-Melo et al. Cell, 2020
	NMC2019-nCoV02 virus	i.n	10^5.5^ TCID_50_	Viral replication in respiratory tract, transmission to naive ferrets; acute bronchiolitis	Kim et al. Cell Host Microbe, 2020
*Mink*					
	SARS-CoV-2/HRB25/human/2020/CHN	i.n	5 × 10^6^ PFU	Viral replication in upper and lower respiratory tract; transmission to naive minks, pulmonary lesions	Shuai et al. Natl. Sci. Rev., 2021
*Mice*					
	2019n-CoV/USA_WA1/2019	i.n	2.5 × 10^4^ PFU	Viral replication in the lung and other organs, severe lung inflammation with cell infiltration, lethality	Winkler et al. Nat. Immunol., 2020
	2019 n-CoV/USA_WA1/2020	i.n.; i.n. + i.v	10^5^ FFU	High viral titers in the lung, lung pathology	Hassan et al. Cell 2020
	COVID-19 patients in Guangzhou and in Washington state (Accession numbers: MT123290, MN985325.1)	i.n	1 × 10^5^ PFU	Pneumonia, pulmonary pathology, and high-titer virus replication in lungs	Sun et al. Cell, 2020
	USA-WA1/2020	i.n	3 × 10^7^ PFU	High viral titers in the lungs, pneumonia, inflammatory cell infiltration	Israelow et al. bioRxiv. 2020

The SARS-CoV-2 isolate, infection route, dosage, and main results are shown. *i.n.* *intra nasal*, *i.t.* *intra tracheal*, *i.v.* *intra venous*, *TCID*_*50*_ Tissue Culture Infection Dose 50, *PFU* Plaque Forming Unit, *FFU* Focus Forming Unit, *dpi* days post-infection

### Non-human primates

Among non-human primates, the most prominent models for COVID-19 research include african green monkeys (*Chlorocebus aethiops*), rhesus macaques (*Macaca mulatta*), and cynomolgus macaques (*Macaca fascicularis*). SARS-CoV-2 infection studies showed that all three species were susceptible to the virus, with rhesus macaques showing the highest susceptibility [17–19]. For this, most animals were infected intranasally or intratracheally with a dose ranging from 1.1 × 10^4^ to 2.6 × 10^6^ TCID_50_ (Tissue Culture Infectious Dose 50) [19–23].

After infection, viral replication in the upper and lower respiratory tract was detected, and disease patterns ranging from non-symptomatic up to moderately severe were observed [19, 21, 24]. In comparison, cynomolgus macaques showed the mildest disease patterns, with virus shedding from the nose and throat. They also showed alveolar damage, but did not develop clinical symptoms [19]. Rhesus macaques developed interstitial pneumonia after infection and viral RNA could be isolated from the respiratory tract. Additionally, infiltration of monocytes and lymphocytes in the alveoli was shown [25]. Despite a clear detectable lung injury, also for this model, no clear clinical symptoms could be detected [18]. African green monkeys (AGM) showed a more robust SARS-CoV-2 replication, with a distinct respiratory impairment, inflammation, and coagulopathy in affected tissues. Unlike the other two models, the AGM showed mild clinical symptoms [17].

These studies show that the non-human primate models reflect some of the COVID-19 disease patterns seen in humans, but do not mimic it completely. Rhesus and cynomolgus macaques may provide important models for non-symptomatic up to mild SARS-CoV-2 disease models. African green monkey infections are slightly more comparable with severe COVID-19 symptoms in humans than the other non-human primate models. This animal model enables investigations concerning the host defense against SARS-CoV-2 and the evaluation of medical countermeasures [17]. Non-human primates in general provide an animal model that is phylogenetically quite close to humans. However, major limitations of non-human primates are the much higher costs and availability of these animals compared to smaller species. An additional point is the limited BSL-3 laboratories for handling non-human primates in SARS-CoV-2 studies.

### Syrian hamsters

The Syrian hamster model (*Mesocricetus auratus)*, already used in studies for SARS-CoV and also for Influenza [26, 27], provides a further model for COVID-19. Several infection studies could prove the susceptibility of hamsters to a SARS-CoV-2 infection [28, 29]. Intranasal challenge with doses from 10^4^ to 10^5^ TCID_50_ of SARS-CoV-2 leads to mild [28] up to moderately severe [30] disease pattern with weight loss, respiratory distress, and lethargy. Additionally, histopathologic changes and high viral load in the lung could be detected in the hamster model. Despite lacking clinical manifestation of the disease, hamsters also develop more enduring disease outcomes, including evidence of myocarditis of the heart and tubular inflammation in the kidney. After SARS-CoV-2 infection, hamsters revealed neutralizing antibodies in serum, and inflammatory cytokine production could be confirmed [30]. Interestingly, hamsters showed viral clearance in the lungs at day 7 post-infection and the animals recovered after infection. Transmission experiments also showed effective transmission between hamsters resulting in similar disease patterns.

The hamster infection model showed similar pathological changes in the upper and lower respiratory tract, with lung inflammation and alveolar damage like in humans. The rapid viral clearance and recovery of hamsters after infection opens new ideas for understanding the underlying immune mechanisms, which provides interesting approaches in this animal model for the development of therapeutic countermeasures against SARS-CoV-2 [28]. These results were relatable to the prolonged disease patterns in patients with post-infection symptoms [31]. Additionally, the well-described transmission of this virus from infected to naïve hamsters by aerosols [28, 30] also enables efficient studies to elucidate the detailed mechanisms during transmission in humans. The susceptibility alongside the quick and favorable economic conditions make the syrian hamster a good model for COVID-19 studies. Nevertheless, the limited amount of hamster-specific antibodies and established assays to thoroughly investigate the pathogenesis and the immune response after infection and also after vaccination still poses a problem in this animal model.

### Ferrets

Ferrets (*Mustela putorius furo*) were already described as a good model for respiratory diseases [32] and are now also used for SARS-CoV-2 challenge experiments. Studies revealed that ferrets are susceptible to SARS-CoV-2 after intranasal infection with doses ranging from 5 × 10^2^ to 5 × 10^6^ PFU (Plaque Forming Unit) [33]. However, the challenge resulted in undetectable up to mild disease patterns. In addition to mild clinical disease symptoms, including lethargy and sneezing, the virus replication was restricted to the upper respiratory and gastrointestinal tract [34] and no viral RNA could be detected in other organs. Pathological examinations of the ferrets showed lymphoplasmacytic perivasculitis and mild peribronchitis, but no severe disease or lethality was observed [35]. Furthermore, studies could show that SARS-CoV-2 can be efficiently transmitted between ferrets [36].

Even though ferrets develop rather mild disease symptoms [33] and the replication is restricted to the upper respiratory tract, this animal model has been established for therapeutic developments and the investigation of the mechanisms underlying mild COVID-19 courses known from humans [37]. Since SARS-CoV-2 infection in ferrets is restricted to the upper but not to the lower respiratory tract, this animal infection model provides research opportunities for the mechanisms preventing organs from being infected, which could also be helpful in therapeutic development. Additionally, they also provide a good model for transmission studies of SARS-CoV-2 [36].

### Minks

Minks (*Neovision Vision*) received important media publicity in summer 2020, being the first farming animal species which was able to infect humans [38]. Minks are used as farming animals for fur production on a large scale in many countries and several farms reported infected and dying animals in 2020 [39, 40]. The susceptibility to SARS-CoV-2 and the transmissibility to humans could make them a new reservoir and a high-risk factor [41]. Thus, the underlying mechanisms of the pathogenesis and transmission in minks should be revealed. Studies could again confirm the susceptibility of minks to SARS-CoV-2 infection in which the animals were intranasally challenged with 5 × 10^6^ PFU of the virus. SARS-CoV-2 replication in the nasal turbinates, and the upper and lower respiratory tracts were detected. The experiments also revealed that the animals are able to transmit the virus and cause infections in other minks and also humans. They developed a severely impaired lung and olfactory function, as severe interstitial pneumonia and perivasculitis were seen after infection [42].

As they show some disease patterns like those seen in humans, this species is also considered to be a suitable animal model for COVID-19 studies. Furthermore, the ability of minks to transmit the virus to humans makes them an important target for transmission experiments. Besides the fact that minks and ferrets are both mustelids, ferrets show milder symptoms and no viral replication in the lungs [35]. Future studies have to elucidate the exact mechanisms and molecular differences leading to this divergence. The severity of the pathogenesis in minks could make them a much more suitable model for the severe course seen in humans than ferrets, which are more likely to provide a platform for milder disease mechanisms [42, 42].

### Mice

One of the most prominent species used in research are mice (*Mus musculus*). Mice are one of the best-established animal models, and their advantages are good availability, affordability, and easy usage in several different study settings. Nonetheless, the murine ACE2 (mACE2) receptor does not interact with the spike protein, which makes mice not susceptible to a SARS-CoV-2 infection. For this reason, several mice have been used to develop promising mouse models for COVID-19, including transient and transgenic hACE2 expression models as well as mouse-adapted SARS-CoV-2 approaches. These COVID-19 mouse models will be discussed in more detail in the following section.

## Mouse models for COVID-19

Mice as a laboratory animal model have the advantage of a variety of available immunologic reagents and assays to assess immune responses in scientific investigations. They provide several different read-outs with a less amount of time for establishment. Furthermore, the reproduction rate, cost-efficiency, and feasibility, while working and handling mice in the laboratory, make them a desirable animal model. The clear genetic background and also the tools for modification are one major advantage compared to other animal models.

However, a few differences in the relevant amino acid sequence of the murine ACE2 (mACE2) compared to the human receptor (hACE2) prevent the cellular uptake of SARS-CoV-2 in murine cells [43, 44]. Implementing modifications, either on the virus or on the mouse breeds, generates a susceptible mouse study system for infection and vaccination studies while providing several well-established methods and reagents to examine all different kinds of details concerning COVID-19.

Since SARS-CoV also enters the host cells via interaction with the hACE2 receptor [13], several modified mouse strains [45] and mouse-adapted viruses have already been generated [46–48]. Thus, the expression of this receptor in mice was already achievable during SARS-CoV studies. SARS-CoV first emerged in 2002 in China and was designated as a “severe acute respiratory syndrome – corona virus” [49]. SARS-CoV caused severe atypical pneumonia, including fever, myalgia, and coughing in humans. Severely diseased patients also developed lymphopenia and liver dysfunction, followed by lethality in several cases [50, 51]. Compared to SARS-CoV-2, it had a higher mortality rate but was less transmissible [52].

Several approaches were used to stimulate the susceptibility of mice to SARS-CoV-2 for COVID-19 pre-clinical studies including the already established mouse models from SARS-CoV research. The two major approaches are, on the one hand, the usage of modified mouse strains and, on the other hand, the modification of the virus itself.

For the first approach, several modified mouse strains were already generated using different types of biotechnology to make the human ACE-2 receptor available in mice. The transient expression of hACE2 using viral vector-based delivery is mostly performed using adenoviruses (AdV-hACE2) or adeno-associated viruses (AAV-hACE2), which deliver the hACE2 receptor and thus guarantee the susceptibility of mice to SARS-CoV-2 [53]. In contrast to that stand the transgenic mouse lineages with a permanent expression of the hACE2 receptor. The similarity of all those mouse models is the expression of hACE2 in the respiratory tract, which guarantees an infection with SARS-CoV-2 and further mimics certain disease patterns of COVID-19 seen in humans. Given the complex pathogenesis of SARS-CoV-2 with the different disease patterns in humans, those mouse models may highlight several opportunities for pre-clinical SARS-CoV-2 studies. The following section will discuss the different transient and transgenic mouse models with their most important features, which are also presented in Table 2.

**Table 2:** Mouse models for COVID-19. Listed are different mouse models with transient or transgenic hACE2 expression involved in SARS-CoV-2 infection studies.

Infection studies									
Breed	Genetic Background	hACE2 Delivery/Expression	Advantages	Disadvantages	SARS-CoV-2 Isolate	Infection Route	Dosage	Results	Reference
Transient hACE2 Expression									
(AdV-hACE2)	BALB/c	Replication-defective adenoviruses encoding human ACE2 via intranasal administration (2.5 × 108 PFU, i.n.), 5 days prior infection			2019 n-CoV/USA_WA1/2020	i.n.; i.n. + i.v	10^5^ FFU	High viral titers in the lung, lung pathology	Hassan et al. Cell [57]
Adenovirus-mediated delivery of hACE2	C57BL/6 J; WT BALB/cJ	Ad-hACE2 at doses of 2.5 × 108 PFU, 1.0 × 108 PFU or 7.5 × 107 PFU, i.n	Quick adaptation, usable on different genetic backgrounds and combination with transgenic mice, accessibility	Only transient hACE2 expression, equal expression between mice not guaranteed, possible side-effects of vector virus	USA-WA1/2020	i.n	1 × 10^4^ PFU	Viral replication to low titers in nasal turbinates and lung, no clinical signs of infection	Rathnasinghe et al. Emerg. Microbes Infect. [58]
Ad5-hACE2-sensitized mice	BALB/c and C57BL/6	2.5 × 108 FFU of Ad5-ACE2 i.n. 5 days prior infection			COVID-19 patients in Guangzhou and in Washington state (Accession numbers: MT123290, MN985325.1)	i.n	1 × 10^5^ PFU	Pneumonia, pulmonary pathology, high-titer virus replication in lungs	Sun et al. Cell 2020
hACE2-AAV mouse model	C57Bl/6 (B6J)	Adeno-associated virus 9 encoding hACE2, into trachea 5 days prior infection			USA-WA1/2020	i.n	3 × 10^7^ PFU	High viral titers in the lungs, pneumonia, inflammatory cell infiltration	Israelow et al. bioRxiv [59]
Transgenic hACE2 Expression									
K18-hACE2	C57BL/6 J	hACE2 expression under the human keratin 18 promotor (K18)	Permanent expression of hACE2, uniform expression between mice	Time consuming for generation, restricted availability, mice have to be generated again for different genetic backgrounds	USA-WA1/2020	i.n	1 × 10^4^ PFU	High virus titers in the nasal turbinates, lung and brain; high lethality; cytokine/chemokine production	Rathnasinghe et al. Emerg. Microbes Infect. [61]
					2019n-CoV/USA_WA1/2019	i.n	2.5 × 10^4^ PFU	Viral replication in the lung, brain and other organs, severe lung inflammation with cell infiltration, lethality	Winkler et al. Nat. Immunol [63]
					Hong Kong/VM20001061/2020	i.n	8 × 10^4^ TCID_50_	Clinical symptoms, high viral titers in the lungs, altered lung histology, interstitial inflammatory cell infiltration	Moreau et al. Am. J. Trop. Med. Hyg. [62]
HFH4-hACE2	C3H, C57BL/6	hACE2 expression under a lung ciliated epithelial cell-specific HFH4/FOXJ1 promoter			SARS-CoV-2 (IVCAS 6.7512)	i.n	7 × 10^5^ TCID_50_	Interstitial pneumonia, virus detected prominently in the lung and additionally in eye, heart and brain	Jiang et al. Cell [65]
AC70 CAG-hACE2	C3H × C57BL/6	hACE2 expression driven by the ubiquitous CAG promoter			SZTH-003 (323P); SZ454 (323L)	i.n	1 × 10^4^ TCID_50_	High and quick lethality, multiple inflammatory cytokines in the lungs, activated RIPK1 was also found in the lungs	Xu et al. Cell Res. [67]
hACE2 mice	–	hACE2 expression driven by the murine ACE2 promotor			BetaCoV/Wuhan/IVDC-HB-01/2020|EPI_ISL_402119	i.n	10^5 ^TCID_50_	Virus replication in the lungs, interstitial pneumonia with infiltration of lymphocytes into the alveolar interstitium	Bao et al. Nature [64]
hACE2	C57BL/6	insertion of hACE2 sequence by CRISPR/Cas9			BetaCoV/wuhan/AMMS01/2020	i.n	4 × 10^5^ PFU	High viral loads in lung, trachea, and brain, interstitial pneumonia and elevated cytokines in aged annimals	Sun et al., Cell Host Microbe [58]

The genetic background, hACE2 delivery/expression in the mouse breed, advantages and disadvantages, SARS-CoV-2 isolate, the infection route, the dosage, and the main results are shown. i.n.: *intra nasal*, i.v.: *intra venous*, *TCID*_50_ Tissue Culture Infection Dose 50, *PFU* plaque- forming unit, *FFU* focus forming unit

## Mouse models based on transient hACE2 expression

The technique of transient expression uses the vector virus-mediated delivery of a target sequence in the animal. Recombinant adenovirus vectors are a well-established tool in molecular biology, for example, as a promising delivery vector system for transgene expression in vitro and in vivo. These viruses are well suited for expression delivery in animals, since the deletion of their E1 gene enables a replication deficiency [54], and their genome is easy to manipulate for the delivery of different target sequences [55]. Additionally, this method provides the opportunity to include a reporter gene, which indicates a successful transduction. This is shown by coronavirus studies with an adenovirus-ACE2-mCherry construct [56]. Studies with adenovirus-mediated expression used in COVID-19 disease research will be further discussed.

### Adenovirus vectors

There are mainly two different techniques for the viral vector-mediated hACE2 expression in mice. The delivery of the hACE2 is performed equally, but researchers use either replication-deficient adenoviruses (AdV) [57] or adenovirus-associated viruses (AAV) as a vector system [58]. Both models were already used in SARS-CoV-2 infection studies.

Expression of the human ACE2 in the lung was generated with the AdV technology by an intranasal application of 2.5 × 10^8^ PFU of the vector virus delivering the hACE2 sequence (AdV-hACE2 [57] or Ad5-hACE2 [58]) or an intratracheal application of 5 × 10^8^ PFU of the adenovirus-ACE2-mCherry [56]. The application in studies using adenovirus-associated viruses (AAV) was also performed in the trachea [59]. After 3–5 days, the mice were intranasally infected with 1.5 × 10^4^ TCID_50_ [56], 1 × 10^5^ PFU [58], or 3 × 10^7^ PFU [59] of SARS-CoV-2. All studies could show that the mice were susceptible to SARS-CoV-2 and the investigators observed high viral titers in the lungs followed by pneumonia [58] and inflammatory cell infiltration [59]. Antibody treatment studies could underline the relevance of these countermeasures against COVID-19 and additionally the suitability of adenovirus vector-hACE2 mouse models for investigations of therapeutic antibody candidates [57].

To compare the two presented adenovirus strategies, adenovirus-associated viruses (AAV) have several advantages. The viral replication in the lung was lower in mice administered with adenovirus-associated viruses (AAV) than those with the replication-deficient adenovirus model [59]. Furthermore, AAV have a lower immunogenicity, which is an important factor while performing immunologic studies after infection. Additionally, the expression of adenovirus-associated viruses is more durable, which allows more experiments during this longer time period [60].

### Benefits and limitations of transient mouse models

The expression via virus vectors, like adenoviruses, represents a good available and cost-efficient method with application opportunities for almost any mouse strain. Especially, the combination with knock-out mice provides a broad spectrum for different experimental designs, which enables more rapid research and development of countermeasures against COVID-19. Especially, insights into the detailed immune responses after infection could probably be investigated using this method [59]. This brings an advantage for the vector-mediated expression models compared to the transgenic mouse models. However, just a transient and thus temporary expression of the target protein in the animal can be achieved. In contrast, fully transgenic mice express the hACE2 receptor permanently. Virus vectors can also induce mild symptoms or immune responses themselves, which is important for consideration while evaluating the clinical outcome during research studies. Additionally, the adenovirus-mediated expression cannot exclude a variation in the expression of the receptor and also differences in the tissue distribution between the mice. A homogeneous expression and a defined tissue distribution are more likely to be guaranteed using the transgenic-modified strains [57]. Moreover, studies comparing a transgenic model (K18-hACE2) with the adenovirus-hACE2 modified mice revealed lower viral titers in the lung, no titers in the brain, and also no clinical symptoms for the AdV-hACE2 mice after SARS-CoV-2 challenge [57]. In general, mice modified with AdV-hACE2 or AAV-hACE2 provide easy adaptable experimental designs and also serve as a good model for antiviral therapy, studies concerning the immune response after infection, and even vaccination studies.

## Mouse models based on transgenic hACE2 expression

In contrast to the previously described transient expression, the development of a transgenic mouse model requires more effort but enables the permanent expression of the hACE2 receptor. Different transgenic mouse lineages are discussed for COVID-19 studies in the following.

### K18-hACE2 mice

The K18-hACE2 mouse model is a transgenic lineage on the C57BL/6 background, which expresses the human angiotensin-converting enzyme 2 (hACE2) in several different tissues and especially airway epithelial cells. This expression enables the uptake of SARS-CoV-2 into the cells in the respiratory tract where the typical COVID-19 symptoms normally begin. These mice already showed effective results in SARS-CoV infection studies [47]. This makes the K18-hACE2 lineage a promising animal model for SARS-CoV-2 infection studies, due to the similarities of SARS-CoV and SARS-CoV-2 concerning their affinity to the hACE2 receptor. For SARS-CoV studies, K18-hACE2 mice were intranasally infected with 7.6 × 10^6^ PFU, which resulted in a rapid lethal infection with viral replication in the lung and spread to the brain. Furthermore, also central nervous system disease with high inflammatory cytokine levels was detected [47]. Compared to the human disease course, the SARS-CoV pathogenesis in the K18-hACE2 mice showed an extended clinical disease with the affection of the brain.

The intranasal infection with SARS-CoV-2 (1–2.5 × 10^4^ PFU) of the K18-hACE2 mice resulted in severe infection of the lungs, and also spreading events into other organs like the brain [61]. High viral titers could be detected in the lung and brain, which led to severe disease and death. The infiltration of immune cells (monocytes, neutrophils and activated T cells) and the secretion of pro-inflammatory cytokines resulted in a severely impaired function of the pulmonary areas [62]. This cell recruitment was also seen in BAL (bronchoalveolar lavage) samples from severe COVID-19 patients [63]. The infection of the lung and other organs mirrors the expression of the hACE2 receptor in many different tissues in the K18-hACE2 model.

The severe disease which followed after SARS-CoV-2 infection in K18-hACE2 mice is an important feature of this animal model, which reflects the rather severe course of COVID-19 in comparison to the mild and moderate pathogenesis shown by other mouse models [57, 64, 65]. Studies revealed many similarities with the severe courses of COVID-19 in humans. Nevertheless, an exact mimicking of the disease pattern like in humans could not be achieved, especially while looking at the brain viral titers, resulting in encephalitis, which was not seen in humans.

The K18-hACE2 lineage is a well-suited model for SARS-CoV-2 infection and vaccination studies as well as for analyzing the resulting immune responses [66]. However, the K18-hACE2 model also has its limitations, since the expression of the receptor and the appearance of the K18-promotor are not physiological. Therefore, the expression of the receptor and thus the tissue tropism of SARS-CoV-2 in this animal model could differ from natural ACE2 occurrence. The severe disease course in the K18-hACE2 mice after SARS-CoV-2 infection does not display the most commonly seen pathogenesis in humans. Nevertheless, this mouse model is well suited for gathering more knowledge about several severe human cases [63].

### AC70 mice

The AC70 mice were created with a mixed genetic background (C3H and C57BL/6) and express the human ACE2 receptor under the CAG promoter, consisting of the cytomegalovirus immediate–early (CMV-IE) gene enhancer and the chicken β-actin promoter. They already showed susceptibility to SARS-CoV with viral growth and tissue pathology [48], and therefore were also proved suitable for SARS-CoV-2 studies. Infection studies with an infectious dose of 1 × 10^4^ TCID_50_ revealed susceptibility of those mice to SARS-CoV-2 with 100% mortality already 5 days after virus inoculation [67]. Compared to the K18-hACE2 mice, this model showed a much higher mortality rate [63] with the development of encephalitis in several mice. Due to this clinical manifestation, the AC70 lineage is used to study in vivo therapeutics against COVID-19 [67]. A strain with such a high and quick morbidity is well suited for such studies, since an improvement in symptoms is clearly obvious. However, concerning the investigations into the exact disease patterns and the pathogenesis of SARS-CoV-2, this model might not be as well suited as other mouse models.

### hACE2 mice

Another transgenic lineage, hACE2 mice, was produced using the human ACE2 sequence driven by the murine ACE2 promotor. This construct was microinjected into the pronuclei of fertilized mice ovaries. The expression of the human ACE2 receptor mainly concerned the lung, heart, kidneys, and intestine. Infection with 1 × 10^5^ TCID_50_ of SARS-CoV-2 resulted in mild to moderate disease symptoms, but no lethality was detected [64]. The infected mice showed weight loss and virus replication in the lungs, resulting in moderate interstitial pneumonia. Alongside the recruitment of macrophages and lymphocytes into the lungs, specific antibodies were detected after infection. Compared to K18-hACE2 mice, the hACE2 model showed less severe disease patterns, which makes them more suitable for rather mild to moderate COVID-19 infection studies [63, 64]. Similar to other transgenically generated mouse lineages, the availability of this strain could be a limitation of this model. The development of these mice is time-consuming and requires complex molecular techniques. However, this mouse lineage could provide a suitable model for antiviral therapeutic and also vaccine development [64].

### HFH4-hACE2 mice

The HFH4-hACE2 mice have a mixed genetic background (C3H, C57BL/6) and express the human ACE2 receptor under the HFH4/FOXJ1 promotor (lung ciliated epithelial cell-specific promotor) [68]. Those mice express the hACE2 receptor in high levels in the lung but additionally also in the brain, liver, kidney, and gastrointestinal tract. Previous investigations could already confirm the susceptibility of this mouse lineage to SARS-CoV [68]. Infection studies with an inoculation of 7 × 10^5^ TCID_50_ of SARS-CoV-2 in the HFH4-hACE2 mice resulted in different disease outcomes. Symptoms like interstitial pneumonia and pathologic changes in the lung and the heart after infection led to lethality of several mice. Additionally, inflammatory cell infiltration into the lung and high viral titers in the brain were observed in those mice. Viral RNA could be found prominently in the lung but also in the brain, eye, and heart of some mice. A gender-specific difference could be observed, while male mice showed a higher mortality than female ones, which reflects the situations also seen in humans. Surviving mice showed low titers of neutralizing antibodies after infection and were used in a re-challenge experiment where they showed resistance to a high dose of SARS-CoV-2, by only generating mild pneumonia. The HFH4-hACE2 mouse model reflected some parts of the COVID-19 disease with mild to severe courses. This enables several opportunities for research usage with these mice. The inconsistent infection efficacy resulting in the highly different disease outcome could, on the one hand, reflect a good variance for testing different levels of severity in one model. Nonetheless, on the other hand, the comparison between the mice and a solid result evaluation could prove more difficulties than in mouse models, like the K18-hACE2 mice with a more uniform disease outcome [63, 65]. This could make the HFH4-hACE2 lineage a suitable model for vaccination and therapeutic studies. Nevertheless, this model showed lethal encephalitis, which does not reflect the exact disease patterns of COVID-19 seen in humans [65].

### Knock-in mice

A different technique is shown by the knock-in strategy using the CRISPR/Cas9 method. The full cDNA of the human ACE2 receptor was inserted into the exon 2 of the murine ACE2 gene, which resulted in a disruption of this mouse gene with no further expression of the mouse but the appearance of the human ACE2 receptor. The construct was injected into zygotes of C57BL/6 mice, and the correct insertion was confirmed by PCR. Young and old mice were tested in SARS-CoV-2 infection studies with a dose of 4 × 10^5^ PFU. High viral loads in the lung and brain were detected in both groups, whereas just the old mice showed significantly decreased body weight levels and also more severe histological disease outcomes. Nevertheless, no severe disease patterns and no lethality occurred after SARS-CoV-2 infection [69]. The disease outcome using the CRISPR/Cas9 modified mice makes them a suitable model for rather mild COVID-19 disease research. The results concerning the age-dependent disease severity also open up important study areas for gaining better knowledge concerning the more severe pathogenesis of SARS-CoV-2 in the elderly.

### Benefits and limitations of transgenic mouse models

Several transgenic mouse lineages are available, which express the hACE2 receptor. Even if the generation of those mouse models is more time-consuming than the one of transient modes, all transgenic mice are permanently susceptible to SARS-CoV-2 and provide a stable genetic background for infection or vaccination studies. However, depending upon the promotors which are used, the expression of hACE2 within the tissues varies between the different models, leading to different replication in the organs and cell types and thus resulting in different disease courses [47, 48, 64]. The clinical disease outcome varies in the different SARS-CoV-2 mouse models due to different expression levels of the human ACE2 in cells and tissues. Nevertheless, the stable and permanent hACE2 expression in transgenic mice highlights several opportunities for using this model for pre-clinical SARS-CoV-2 studies.

### Mouse-adapted SARS-CoV-2

The generation of mouse-adapted virus strains provides a good additional system for SARS-CoV-2 infection studies. Such a virus strain already showed promising data during investigations for SARS-CoV [46], which provides a good basis for the generation of a mouse-adapted SARS-CoV-2 strain. Using reverse genetics, the modification of the spike protein, which binds to the hACE2 receptor, can be achieved, which guarantees an affinity to the murine ACE-2 receptor.

The intranasal infection of BALB/c mice with 1 × 10^5^ PFU of a SARS-CoV-2 virus, modified by site-directed mutagenesis, resulted in a susceptibility of the mice with impaired lung function ranging from mild to moderate–severe disease symptoms [70]. Another study showed the generation of a mouse-adapted SARS-CoV-2 (MASCp6) by passaging a clinical isolate of SARS-CoV-2 six times in the respiratory tract of aged BALB/c mice. This modification generated five mutations in the genome of the virus, which increased the susceptibility of murine cells. This virus showed more affinity to the murine cells by showing an increased infectivity in the mouse lungs. Studies could confirm the replication efficacy of MASCp6 in the lower respiratory tract of the mice with subsequent interstitial pneumonia and inflammatory immune responses. High amounts of viral RNA were found in the lungs and also in the trachea, heart, liver, spleen, and brain. However, this modified virus could not reveal clinical symptoms or mortality in the mice [71].

### Benefits and limitations of mouse-adapted SARS-CoV-2 strains

Mouse-adapted virus strains provide the ability to use several commercially available mouse lineages or even specific knock-out mice for the infection studies. Thus, this strategy is more feasible and less time-consuming than generating modified mouse lineages. This system also does not interfere with a non-physiological expression of hACE2 in the murine cells, like in modified mice. Furthermore, with the possibility of using immune-competent mice as in the aforementioned study, the research question can be analyzed in much more detail [71]. However, for studying the pathogenesis of SARS-CoV-2, the exact virus causing COVID-19 in humans could provide a more realistic basis for investigations. The properties of the virus and the resulting clinical outcome can vary in different ways from the non-modified SARS-CoV-2 and thus diminish the transferability of the results. Nevertheless, those modified virus strains used in mice can provide a different important research field concerning upcoming variants of SARS-CoV-2, which are modified in the receptor-binding domain.

### SASRS-CoV-2-specific immunity: T cells

Characterizing the immunity after a SARS-CoV-2 infection is important for the understanding of the pathogenesis of SARS-CoV-2. The immunologic components involved in the enhancement of COVID-19 and the correlates of protection after vaccination have to be elucidated. This will enhance the development of therapeutic agents and efficiently protecting vaccines, since SARS-CoV-2 reactive memory T cells can be used to monitor long-term immunity after natural infection [72] and vaccination [73].

T cells are one major component in the adaptive immunity and function in different ways, from supporting the production of antibodies by B cells to cytotoxic reaction against virus-infected host cells [74]. T cells play an important role in viral clearance in terms of induced cell death of infected cells but also after infection as a part of the protective memory, which is also achieved by vaccinations [75]. Additionally, their involvement in the bronchus-associated lymphoid tissue (BALT) is especially important during infections of the respiratory tract, like in COVID-19 [76]. However, like most of the cells, those immunologic key players can also be detrimental to the outcome of disease [77]. While heavily infiltrating into tissues and organs with an overshooting cytokine secretion and cytotoxic activity, T cells can be the cause of a much more enhanced and severe disease course [78, 79]. Even the involvement after vaccination is quite different, resulting in varying durable protection efficacy [75] and side-effect reactions, like, for example, T-cell-mediated hypersensitivity [80] after vaccination.

Alongside T cells, several other immune cells and immunologic components are hypothesized to be involved in protection and also enhancement during COVID-19 disease. For instance, neutralizing antibodies (nAB) are one major part of the immune protection established after infection or vaccination. nAB bind efficiently to the virus particle and thus contribute to the virus neutralization [81]. Not all patients recovering from COVID-19 produce detectable levels of SARS-CoV-2-specific antibodies and even individuals with a mild disease course revealed low titers [82], which were often followed by a rapid decline in antibodies in those and also asymptomatic patients [83, 84]. However, the T-cell repertoire is considered to provide a more durable protection after infection [85–87]. Recent studies already revealed that SARS-CoV-2-specific T cells are prominent in acute phase COVID-19 patients and interrelate with the severity of the disease [88], which could also enable production opportunities of SARS-CoV-2 reactive T cells as a treatment option [89]. Regarding upcoming variants of SARS-CoV-2, those virus particles are more likely to escape from the previously secreted neutralizing antibodies. However, T cells are probably more capable of sustaining effectivity against different variants of the virus, resulting in a cross-interaction of the specific T cells with SARS-CoV-2 variants [86, 90]. Analyzing the appearance and mechanisms of cross-reactive T cells could also enhance the design of trial investigations for further COVID-19 vaccines [91, 92].

**Fig. 1:**
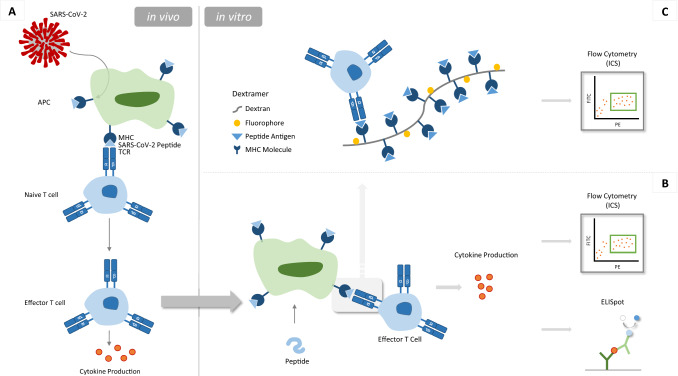
Analysis of SARS-CoV-2-specific T cells. The analysis of SARS-CoV-2-specific T cells is an important tool to contribute to a better understanding of the SARS-CoV-2 pathogenesis and for developing therapeutic countermeasures and vaccines against COVID-19. **A** During an infection, antigen-presenting cells (APC) present peptides of SARS-CoV-2 to naive T cells. Naive T cells with a matching T cell receptor (TCR) bind the MHC–peptide complex and proliferate and maturate to effector T cells. One effector function is the production of inflammatory cytokines like IFN-γ (Interferone-gamma). The occurrence and the amount of those specific T cells against SARS-CoV-2 can be analyzed using different techniques. **B** SARS-CoV-2 specific T cells can be again stimulated in vitro and the resulting cytokine production analyzed in two read-outs. For the stimulation, PBMC or splenocytes are isolated from the mouse containing the specific T cells and the also important APCs (antigen-presenting cells). This cell suspension is then incubated with peptides from different protein parts of the SARS-CoV-2 virus particle. The APCs in the mixture then engulf the peptides, and process and present them on their surface via the MHC molecules. Potential specific T cells bind to this complex and further secrete cytokines (especially IFN-γ, Interferone-gamma) upon activation. IFN-γ can be detected either using an intracellular immunostaining (ICS) and analysis in a flow cytometer or in an ELIspot assay. **C** SARS-CoV-2 specific T cells can also be directly labeled using dextramers. These dextran backbones are assembled with 10 MHC-Molecules carrying a peptide of a SARS-CoV-2 protein. This complex can then bind to a specific T cell. For visualization, the structure also binds several fluorophores for the analysis in a flow cytometer. Several other compositions are also available alongside the here shown dextramer structure, like tetramers and monomers carrying fewer of the MHC–peptide complexes but also resulting in a decreased signal intensity in the flow cytometer analysis. **B-C** FITC and PE are representative fluorophores for signal detection in a flow cytometer

To analyze the involvement of T cells in these different immunological situations described above, the appearance of those cells during in vivo experiments has to be analyzed. During infection or vaccination studies, the virus or viral particles are engulfed by antigen-presenting cells (APCs) such as dendritic cells (DCs), and further processed and presented as the MHC–peptide complex on their cell surfaces [74,93]. This complex is presented to T cells, all of these recognizing different kinds of peptides bound to the MHC molecule. The specific but still naïve T cells bind and are activated, resulting in effector T cells, which produce pro-inflammatory cytokines, for example IFN-γ (Interferon-gamma) (Fig. 1A) [93–95]. To analyze the appearance and amount of those specific effector T cells, the epitopes of SARS-CoV-2 recognized by the T cells must be elucidated. Fragments mirroring the exact epitope of the virus can then be presented and reactivate these T cells in vitro. This activation can be visualized using different read-outs. If the exact epitopes are not already known, the usage of peptide pools covering the whole sequence of a specific protein, for example the S-protein of SARS-CoV-2, can help to start map the immune-dominant epitopes, which are recognized by the specific T cells [96–98]. For this, PBMCs (peripheral blood mononuclear cells) are extracted from mice used in the experiment, which contain T cells and also APCs. Combining those cells with different peptide pools, the APCs present the peptides on their surfaces and activate specific T cells (Fig. 1B). The choice of different sub-pools enables statements to be made concerning the region of the protein, which is detected by the specific T cells. The smaller the pools are, the more precisely the exact epitope can be found. Detecting the specific T cells can be analyzed in different read-outs. Using an intracellular immunostaining (ICS) towards the IFN-γ, the cells can be visualized in a flow cytometer [99]. Furthermore, this cytokine production can also be measured in an ELISpot assay (Fig. 1B) [100] and also additionally in interferon-gamma release assays (IGRA) [101]. Analysis using the flow cytometer also provides information about the differentiation of T cell subsets while using antibodies for membrane-immunofluorescence staining simultaneously. However, ELISpot assays can also provide the differentiation between CD4^+^ and CD8^+^ T cells [102] and offer a more sensitive mode of detection, since the IFN-γ secretion by single T cells can be determined. Both techniques using the peptide pools offer well-established methods for epitope mapping and already enable the analysis of specific T cells. If the immuno-dominant epitopes are already elucidated, the dextramer-MHC technology can be used. This technique provides a direct labeling of the specific T cells via the MHC–peptide–T-cell–receptor complex. The dextramers consist of a dextran backbone assembled with 10 MHC-Molecules (both MHC-I or MHC-II molecules are possible) carrying a peptide of a SARS-CoV-2 protein, for example the S-protein [103, 104]. T cells with a matching T cell receptor (TCR) will then bind to this complex. The dextran is also assembled with fluorophores to visualize the cells in a flow cytometer (Fig. 1C). Additional compositions like tetramers and monomers are also available, which carry less of the MHC–peptide complexes. However, a higher signal intensity in the flow cytometer analysis can be achieved using dextramers [104]. The advantage of the dextramer technology is the direct labeling of the specific T cells. Nevertheless, for this method, the immuno-dominant epitopes should be already revealed. Furthermore, the visualization of the INF-γ production confirms the effector function of the T cells, which is not visualized by the dextramer staining.

Studies using convalescent patients already revealed epitopes which are targeted by human T cells [88]. However, while working on pre-clinical studies in animals, the epitopes have to be re-investigated for this exact species. The human studies revealed that the specific T cells mostly targeted the S- but also the N- and M-proteins [88, 105]. Mapping those epitopes in the murine model was already performed using mice expressing the hACE2 receptor (Ad5-hACE2, BALB/c, and C57BL/6 mice). Those investigations revealed epitopes of SARS-CoV-2 which are targeted by CD4^+^ and CD8^+^ murine T cells [106].

The analysis of specific T cells after infection or vaccination might contribute to a better understanding of correlates of protection. This information is very important for the evaluation of candidate vaccines. Nevertheless, additional methods can be used to further elucidate the protective effect of these cells in in vivo infection studies. The depletion of T cells or a subpopulation like CD4^+^ or CD8^+^ T cells enables a statement about the involvement during the immune response [107]. With this technique, the step from correlating to causative component of protection can be made.

### Next-generation mouse models

Since to date, no tested animal model reflects the exact pathogenesis of SARS-CoV-2 infection in all aspects [108, 109], optimizing and adapting established models are still valuable. Given the possibility that this exact outcome will not be possible in an animal species or the achieving thereof will take time, the most important task is to establish a suitable animal model for the specific issues concerning the SARS-CoV-2 pathogenesis and the vaccination and medical treatment studies. The previously discussed mouse models already provide a good basis for studying the SARS-CoV-2 pathogenesis and preventive measures. Their different advantages have to be further elucidated and refined to implement them in those different investigations. Also, with regard to the upcoming variants of SARS-CoV-2 and even new coronaviruses, improving and adjusting those mouse models are of utmost importance. Adapted mouse model systems will then help to gather more knowledge concerning the pathogenesis of SARS-CoV-2 and may also contribute to a better start regarding a possible new epidemic or even pandemic with upcoming variants or even new viruses [85, 87].

Obviously, variations between mice and humans should not be ignored, especially considering the expression of the human ACE2 receptor in the tissues. Since the expression of hACE2 in mice is driven by molecular modification, the tissue and cellular tropism of the virus may not reflect the situation in the human body [47, 61]. Despite limitations, the mouse models described above in general provide a good repertoire for studies concerning COVID-19. The different strains and systems generate several aspects of the disease patterns seen in humans. Even though they cannot reflect the exact symptoms, they constitute a good collection for different aspects of this disease [108]. Regarding the various expressions from asymptomatic, mild, and severe courses of the disease, these mouse models can be used for further studies in COVID-19 research. These mouse models form a strong basis for a future pool of techniques and animal models, which will, when optimized, provide a well-established and efficient working system for investigations into respiratory diseases.

Additionally, other animal species also contribute to COVID-19 research and the development of effective countermeasures. Just to mention a few further species, cats and dogs for example, are also susceptible to SARS-CoV-2 and thus could also be an interesting research field for infection studies [35, 110]. Studies using cats showed none or only mild symptoms after infection [35]. Interestingly, despite the fact that cats as a popular pet are also able to be infected and develop symptoms, the rather asymptomatic course and also close emotional contact with humans makes this animal model a difficult choice for infection studies. The same would apply to dogs. Nevertheless, dogs are currently being used as sniffing dogs for discerning between infected and non-infected patients [111].

Of course, differences between animals and humans cannot be ignored, which makes the right choice of the animal model highly important. This model should mimic the pathogenesis known in humans as close as possible to guarantee a good transfer of the results into the human system. Still, not every well-suited animal model is also a good choice for conducting the planned experiments due to feasibility, reproduction rates, cost-efficiency, and also ethical reasons.
